# Assessment of the Accuracy of High-Throughput Sequencing of the ITS1 Region of Neocallimastigomycota for Community Composition Analysis

**DOI:** 10.3389/fmicb.2019.02370

**Published:** 2019-10-18

**Authors:** Joan E. Edwards, Gerben D. A. Hermes, Sandra Kittelmann, Bart Nijsse, Hauke Smidt

**Affiliations:** ^1^Laboratory of Microbiology, Wageningen University & Research, Wageningen, Netherlands; ^2^WIL@NUS Corporate Laboratory, Centre for Translational Medicine, National University of Singapore, Singapore, Singapore; ^3^Laboratory of Systems and Synthetic Biology, Wageningen University & Research, Wageningen, Netherlands

**Keywords:** Neocallimastigomycota, anaerobic fungi, internal transcribed spacer 1 region, high-throughput sequencing, clone library, size polymorphism

## Abstract

Anaerobic fungi (Neocallimastigomycota) are common inhabitants of the digestive tract of large mammalian herbivores, where they make an important contribution to plant biomass degradation. The internal transcribed spacer 1 (ITS1) region is currently the molecular marker of choice for anaerobic fungal community analysis, despite its known size polymorphism and heterogeneity. The aim of this study was to assess the accuracy of high-throughput sequencing of the ITS1 region of anaerobic fungi for community composition analysis. To this end, full-length ITS1 clone libraries from five pure cultures, representing the ITS1 region size range, were Sanger sequenced to generate a reference dataset. Barcoded amplicons of the same five pure cultures, and four different mock communities derived from them, were then sequenced using Illumina HiSeq. The resulting sequences were then assessed in relation to either the reference dataset (for the pure cultures) or the corresponding theoretical mock communities. Annotation of sequences obtained from individual pure cultures was not always consistent at the clade or genus level, irrespective of whether data from clone libraries or high-throughput sequencing were analyzed. The detection limit of the high-throughput sequencing method appeared to be influenced by factors other than the parameters used during data processing, as some taxa with theoretical values >0.6% were not detected in the mock communities. The high number of PCR cycles used was considered to be a potential explanation for this observation. Accuracy of two of the four mock communities was limited, and this was speculated to be due to preferential amplification of smaller sized ITS1 regions. If this is true, then this is predicted to be an issue with only six of the 32 named anaerobic fungal clades. Whilst high-throughput sequencing of the ITS1 region from anaerobic fungi can be used for environmental sample analysis, we conclude that the accuracy of the method is influenced by sample community composition. Furthermore, ambiguity in the annotation of sequences within pure cultures due to ITS1 heterogeneity reinforces the limitations of the ITS1 region for the taxonomic assignment of anaerobic fungi. In order to overcome these issues, there is a need to develop an alternative taxonomic marker for anaerobic fungi.

## Introduction

Neocallimastigomycota are an important class of strictly anaerobic fungi that are commonly found in herbivore gut ecosystems, particularly that of large mammals. Anaerobic fungi have been most extensively studied in ruminants, where they can increase fiber degradation and feed intake by 7–9% and up to 40%, respectively (Gordon and Phillips, 2005). Due to their potent fiber degrading enzymes (Solomon et al., 2016), anaerobic fungi are also of great biotechnological interest. Eleven anaerobic fungal genera are currently characterized (Edwards et al., 2017; Hanafy et al., 2018; Joshi et al., 2018), with evidence for the existence of more genera from cultivation independent analysis of environmental samples (Liggenstoffer et al., 2010; Nicholson et al., 2010; Kittelmann et al., 2012, 2013). Using the ITS1 region of anaerobic fungi, a taxonomic framework and associated curated database have been developed, which classifies ITS1 sequences to characterized genera and as yet uncultured genus- or species-level clades (Koetschan et al., 2014). This is a valuable resource for the analysis of sequences obtained from environmental samples, particularly when using HTS of barcoded amplicons, which has become the method of choice for determining anaerobic fungal community composition (Liggenstoffer et al., 2010; Kittelmann et al., 2012, 2013). However, it has since been recognized that the forward primer site based within the ITS1 region (primer MN100F) used is not conserved in all anaerobic fungi (Kittelmann et al., 2013; Callaghan et al., 2015).

Due to the lack of conserved priming sites within the ITS1 region for anaerobic fungal specific amplification, primers targeting the more conserved 18S and 5.8S rRNA genic flanking regions are recommended. The primers previously developed for anaerobic fungal specific automated ribosomal intergenic spacer analysis (ARISA), which generate a ∼350–440 bp amplicon, are such an example (Edwards et al., 2008). Based on full-length sequences in the database of Koetschan et al. (2014), the ITS1 region ranges in size from 192–282 bases. Whilst this ITS1 size polymorphism is valuable for ARISA, it is problematic for creating a stable ITS1 phylogeny unless sequence alignments are improved using secondary structure information (Edwards et al., 2017).

Internal transcribed spacer 1 size heterogeneity exists not only between anaerobic fungal pure cultures, but also within them (Edwards et al., 2008). As such, it is perhaps not surprising that within a single culture multiple cloned ITS1 sequences can vary as much as 13% between ITS1 repeats (Callaghan et al., 2015). However, the implication that this has for the interpretation of high-throughput sequencing data generated from pure cultures and environmental samples is not known. The objective of this study was, therefore, to assess the accuracy of HTS of the ITS1 region of anaerobic fungi based on the previously published ARISA primers (Edwards et al., 2008), using anaerobic fungal pure cultures and defined mock communities of different composition and complexity. This is important not only in terms of data quality control, but also to identify issues associated with polymorphism and heterogeneity within the ITS1 region. Following current debate about the value of the ITS1 region for anaerobic fungal analysis (Edwards et al., 2017), the findings of this study will provide a clear evidence base regarding the strengths and limitations of its use as an anaerobic fungal taxonomic marker.

## Materials and Methods

### Pure Cultures and DNA

The five pure culture DNA extracts used in this study were kindly provided by Dr. Tony M. Callaghan and Veronika Dollhofer (Bavarian State Research Center for Agriculture, Freising, Germany), and were obtained as previously described (Dollhofer et al., 2016). *Neocallimastix frontalis* strain RE1 and *Orpinomyces* sp. SR2 (also known as *Orpinomyces* sp. OUS1) were isolated from the sheep rumen (Stewart and Richardson, 1989; Brookman et al., 2000). *Anaeromyces* sp. 28xy was isolated from feces of a Highland cow (Callaghan, 2014). *Piromyces* sp. CaDo16a was isolated from digester sludge of a Bavarian biogas plant (Dollhofer et al., 2017). *Caecomyces* sp. CaDo13a was isolated from rumen fluid of a wild alpine goat (personal communication, Callaghan and Dollhofer). Available ITS region sequence data from one of the five pure cultures, *Piromyces* sp. CaDo16a, is assigned to the species hypothesis code SH1571620.08FU in the UNITE database (Nilsson et al., 2018).

### Clone Library Based Sequencing of Pure Cultures

For each of the five pure cultures an ITS1 reference dataset was created using a clone library approach. A PCR amplicon comprising the partial 18S rRNA gene (∼310 bp), full ITS1 region and partial 5.8S rRNA gene (116 bp) was amplified using the forward primer 5′-CAT CCT TGA TCG GRA GGT CC-3′ (i.e., the AF-SSU reverse primer of Dollhofer et al. (2017) in the forward orientation), and the reverse primer “Neo QPCR Rev” (5′-GTG CAA TAT GCG TTC GAA GAT T-3′, Edwards et al., 2008). PCR was performed in triplicate for each culture using 50 μL reactions containing 1 × HF buffer (Finnzymes, Vantaa, Finland), 1 μL dNTP Mix (10 mM; Promega, Leiden, Netherlands), 2 U of Phusion^®^ Hot Start II High-Fidelity DNA polymerase (Finnzymes), 500 nM of each primer, and 2 ng of DNA. The cycling conditions consisted of an initial denaturation at 98°C for 3 min followed by 40 cycles of 98°C for 10 s, 50°C for 30 s, and 72°C for 30 s, and a final extension at 72°C for 6 min. Successful amplification was confirmed by agarose gel electrophoresis on a 2% (w/v) agarose gel containing 1 × SYBR^®^ Safe (Invitrogen, Carlsbad, CA, United States). A NTC reaction was also performed and generated no PCR product. A pooled PCR product for each of the five pure cultures was purified using HighPrep^TM^ (MagBio Europe Ltd., Kent, United Kingdom), and quantified using a Qubit fluorometer in combination with the dsDNA BR Assay Kit (Invitrogen). PCR products were then A-tailed and cloned using the pGEM-T easy vector system (Promega). Using blue/white screening, transformed white clones were randomly selected (19–20 per pure culture) and sent for Sanger sequencing using both the M13F and M13R priming sites within the vector (GATC-Biotech, Cologne, Germany). The quality of reads was manually verified, and consensus reads prepared for each clone.

### Preparation of Mock Community Template DNA

Four different mock communities (Mock_1 to Mock_4) were prepared by combining the cleaned and quantified PCR amplicons used for clone library preparation. PCR amplicons were used rather than genomic DNA as the *rrn* operon copy number of the cultures used was not known. PCR amplicons were combined based on the amount of DNA, giving a total of 500 ng in a 50 μl volume. Mock_1 was composed of 250 ng of both *N. frontalis* RE1 and *Anaeromyces* sp. 28xy. Mock_2 was composed of 100 ng of each of the five pure cultures. Mock_3 was composed of 250 ng of *N. frontalis* RE1, 100 ng of *Orpinomyces* sp. SR2, 75 ng of *Piromyces* sp. CaDo16a, 50 ng of *Caecomyces* sp. CaDo13a, and 25 ng of *Anaeromyces* sp. 28xy. Mock_4 was composed of 88.89 ng of *N. frontalis* RE1, 10 ng of *Caecomyces* sp. CaDo13a, 1 ng of *Piromyces* sp. CaDo16a, 0.1 ng of *Orpinomyces* sp. SR2, and 0.01 ng of *Anaeromyces* sp. 28xy. Each mock community was prepared in duplicate, and then pooled to minimize variation associated with pipetting. The theoretical composition of each mock community was then determined taking account of the molarity of each culture PCR amplicon in the mock community. This was done by calculating the number of PCR amplicons in the amount of DNA from each culture present in the mock, using the amount of DNA added (as indicated above) and the average of the size of the clones from the corresponding clone library. Percentage relative abundances were then derived from these values for each mock community.

### Illumina High-Throughput Sequencing

Barcoded amplicons comprising the partial 18S rRNA gene (∼130 bp), full ITS1 region, and partial 5.8S rRNA gene (∼31 bp) were generated for the five pure cultures and four mock communities using a 2-step PCR strategy with a Labcycler (SensoQuest, Göttingen, Germany). This preparation was repeated three times, as all samples were independently run in three different libraries (A, B, and C). Furthermore, mock community samples were also sequenced in duplicate within one library A (i.e., A1 and A2).

The first PCR step was performed using the previously published ARISA primers (Edwards et al., 2008) with the addition of UniTag adapters (underlined): Neo 18S For 5′-GAGCCGTAGCCAGTCTGCAATCCTTCGGATTGGCT-3′ and Neo 5.8S Rev 5′-GCCGTGACCGTGACATCGCGAGAACC AAGAGATCCA-3′. PCR was performed in a total volume of 25 μL containing 1 × HF buffer, 1 μL dNTP Mix (10 mM), 1 U of Phusion^®^ Hot Start II High-Fidelity DNA polymerase, 500 nM of each primer, and 2 ng of pure culture or mock community DNA. The cycling conditions consisted of an initial denaturation at 98°C for 3 min followed by 40 cycles of 98°C for 10 s, 58°C for 30 s, and 72°C for 30 s, and a final extension at 72°C for 6 min. Triplicate PCR reactions were prepared for each sample, along with NTC reactions. The presence of PCR products from samples, and their absence in the NTC, was confirmed by agarose gel electrophoresis on a 2% (w/v) agarose gel containing 1 × SYBR^®^ Safe. Pooled triplicate reactions, as well as the negative individual NTC reactions, were then purified using HighPrep^TM^. NTC reactions were further processed and sequenced in the same manner as the samples so that any OTU (Blaxter et al., 2005) clearly associated with any of the NTC reactions could be manually removed during processing of the resulting sequence data.

The second PCR step was then employed to add an eight nucleotide sample specific barcode to the 5′- and 3′- end of the PCR products as previously described (van Lingen et al., 2017). Each PCR reaction, with a final volume of 100 μL, contained 5 μL of the purified first step PCR product, 5 μL each of barcoded forward and reverse primers (10 μM), 2 μL dNTP Mix (10 mM), 2 U of Phusion^®^ Hot Start II High-Fidelity DNA polymerase, and 1 × HF buffer. Amplification consisted of an initial denaturation at 98°C for 30 s followed by five cycles of 98°C for 10 s, 52°C for 20 s, and 72°C for 20 s, and a final extension at 72°C for 10 min. Barcoded PCR products were then purified using the HighPrep^TM^, and quantified using a Qubit in combination with the dsDNA BR Assay Kit. Purified sample PCR products were then pooled in equimolar amounts, with the exception of the purified NTC PCR products which were included based on the maximum volume of purified sample PCR product used in the equimolar pool. Pools then underwent adaptor ligation followed by sequencing on the Illumina HiSeq platform using 300 PE chemistry (GATC-Biotech, Konstanz, Germany, now part of Eurofins Genomics Germany GmbH).

### Theoretical Mock Community Sequence Files

Theoretical fastq files (forward and reverse) for each mock community were created (T_Mock_1 to T_Mock_4) based on the clone library reference data. These files served multiple purposes. The optimal parameters for bioinformatics processing of the anaerobic fungal HTS data were determined using this reference dataset (Supplementary Figure S1). In addition, comparison of the theoretical mock communities to the actual sequenced mock communities (Mock_1 to Mock_4) enabled determination of whether any biases found were likely to be associated with the generation of the HTS data itself, or its subsequent bioinformatics processing. The files were prepared as follows. For each culture, all the cloned sequences from the reference dataset were first aligned using ClustalW version 2.1 (Larkin et al., 2007) and then trimmed *in silico* to generate ends that matched the PCR primers used for the HTS using GeneDoc version 2.6 (Nicholas and Ncholas, 1997). Using the knowledge of the theoretical composition of each mock community (see above), the trimmed sequences for the pure cultures were then combined in the appropriate proportions to create forward and reverse fastq files each containing a total of 200,000 reads. Different unique barcodes were then added to each theoretical mock community, enabling the corresponding fastq files to be processed in exactly the same way as the sequenced mock community samples during bioinformatics processing. No taxonomic information was included in the files as they were processed in exactly the same manner as samples during data analysis. The script used for generating the theoretical mock communities fastq files, as well as the associated theoretical mock community data used in this study, is available at https://gitlab.com/wurssb/gen _fake_mocks.

### Analysis of High-Throughput Sequence Data

Raw Illumina sequence data and theoretical mock community fastq files were processed using NG-Tax (version NGTax-2.jar^1^). Using an open reference approach, NG-Tax defines OTUs as unique sequences that are above a user-defined minimum abundance threshold (Ramiro-Garcia et al., 2016). NG-Tax filters the PE libraries to contain only read pairs with perfectly matching barcodes, with the details of the sample barcodes and library files used in this study provided in Supplementary Table S1. NG-Tax was performed with the following parameters: PE read length 150 bases (as beyond this length the mean read quality scores deteriorated), ratio OTU abundance 2.0, minimum abundance threshold was set at 0.6% (Supplementary Figure S1), identity level 100%, and error correction of 1 mismatch (99.33%). As the PCR amplicon primers used were not within the AF-ITS1 database (version 3.3^2^) used for OTU annotation (which is a requirement for annotation by NG-Tax), an empty database file (emptydb.fasta.gz^3^) was used and the OTUs then subsequently annotated manually.

Fasta files of the OTUs from the NG-Tax generated biom file^4^ were extracted using the script otuseq_export.py^5^. The OTUs were annotated using BLASTN searches against the AF-ITS1 database using default settings with “-num_alignments 10” (BLAST version 2.4.0). For OTUs that could not be annotated by the AF-ITS1 database, BLASTN searches were performed against the NCBI database. Cut-off levels for OTU annotations were determined based on the mean percentage similarities of full-length sequences in the AF-ITS1 database within clade and within genus. These cut-off levels were >98% for clade and >95% for genus. Based on the study of Koetschan et al. (2014), the term clade is defined as a known species or an uncultivated subgroup within a monophyletic lineage that has been identified using secondary structure informed analysis of ITS1 region sequence data. As previously noted by Koetschan et al. (2014), it is not known if some of the uncultivated subgroups represent new species or potentially new genera. The NG-Tax generated biom file was converted to a tab-delimited table to enable OTU annotations to be added. The OTUs that were clearly associated with the NTC samples were also manually removed from the tab-delimited table at this stage. The resulting tab delimited table was then converted back to a biom file^6^.

Plots were created using ggplot2 (Wickham, 2009) in R version 3.4.0. Accuracy of the sequencing of the mock communities was determined by calculating Pearson correlation values (Pearson, 1909) and pairwise weighted UniFrac distances (Lozupone et al., 2011) between the sequenced mock communities and the corresponding theoretical mock community. To test for differences in accuracy between the mock communities, the data (as described above) for all the mock communities was analyzed by ANOVA and a Tukey *post hoc* test performed (Genstat, 19th edition, VSN International Ltd.). Probability values <0.05 were considered to be significant.

### Data Availability

The Sanger sequenced clone library data is deposited in NCBI under the following accession numbers: *N. frontalis* RE1 (MK036660-MK036676), *Orpinomyces* sp. SR2 (MK036677-MK036695), *Piromyces* sp. CaDo16a (MK036696-MK036714), *Caecomyces* sp. CaDo13a (MK036715-MK036728), and *Anaeromyces* sp. 28xy (MK036729-MK036744). The HTS data is deposited in the European Nucleotide Archive under the study accession number PRJEB29131.

## Results and Discussion

### Clone Library Based Analysis of Anaerobic Fungal Pure Culture Taxonomy and ITS1 Size Polymorphism

Pure cultures of five morphologically distinct anaerobic fungal genera were used to generate a reference ITS1 dataset using cloning and Sanger sequencing. The five anaerobic fungi were *N. frontalis* RE1, *Anaeromyces* sp. 28xy, *Orpinomyces* sp. SR2, *Piromyces* sp. CaDo16a, and *Caecomyces* sp. CaDo13a. All five pure cultures generated ribosomal operon fragments (partial 18S rRNA gene, full ITS1 region, and partial 5.8S rRNA gene), which varied in size both within and between cultures (Table 1). This is consistent with previously published ARISA analysis of anaerobic fungal pure cultures (Edwards et al., 2008).

**TABLE 1:** Sequence size and taxonomy of cloned sequences (partial 18S rRNA gene – full ITS1 region – partial 5.8S rRNA gene) generated from anaerobic fungal pure cultures.

**Genus**	**Strain**	**No. of unique sequences^∗^**	**Sequence size (bases)^#^**	**Taxonomic classification (Genus; Clade)^$^**
*Neocallimastix*	RE1	7 (of 17)	681–705 (17)	*Neocallimastix*; *Neocallimastix 1*
*Orpinomyces*	SR2	6 (of 19)	631–632 (18)	*Orpinomyces*; *Orpinomyces 1a*
			637 (1)	*Orpinomyces*; *Orpinomyces 1b*
*Piromyces*	CaDo16a	3 (of 19)	658–660 (19)	NA; NA^+^
*Caecomyces*	CaDo13a	8 (of 14)	625 (1)	*Cyllamyces*; NA
			657 (2)	*Caecomyces*; NA
			658 (11)	*Caecomyces*; *Caecomyces 1*
*Anaeromyces*	28xy	10 (of 16)	671–672 (9)	*Anaeromyces*; NA
			674–675 (7)	*Anaeromyces*; *Anaeromyces 1*

*^∗^Numbers in parentheses indicate the total number of clones. ^#^Numbers in parentheses indicate the number of clones with the taxonomic classification indicated. ^$^NA, not annotated. ^+^In subsequent analysis in this study, sequences from the *Piromyces* sp. CaDo16a were annotated as “CaDo16a; NA.”*

Findings from the BLAST based annotation of the complete ITS1 region of the cloned sequences against the AF-ITS1 database showed that full and consistent annotation at the clade level, for all clones, only occurred with *N. frontalis* RE1 (clade *Neocallimastix 1*). With *Anaeromyces* sp. 28xy, only seven of the 16 clones could be reliably annotated to the clade level (*Anaeromyces 1*). For *Orpinomyces* sp. SR2, 18 of the 19 clones were annotated as clade *Orpinomyces 1a* whilst one sequence was annotated as clade *Orpinomyces 1b*. This raises a question regarding the validity of the sub-division of the *Orpinomyces 1* clade (Koetschan et al., 2014).

None of the *Piromyces* sp. CaDo16a clones could be annotated at either the clade or genus level using the AF-ITS1 database due to having <90.5% identity. Therefore, in this study sequences matching to *Piromyces* sp. CaDo16a were annotated as “CaDo16a; NA.” The low identity of *Piromyces* sp. CaDo16a to other *Piromyces* sequences in the AF-ITS1 database is not entirely unexpected, as this strain has recently been suggested to represent a new clade within this genus based on phylogenetic analysis of its 28S rRNA gene (Dollhofer et al., 2017). With *Caecomyces* sp. CaDo13a, 11 of the 14 clones were annotated as clade *Caecomyces 1*, whereas two of the clones could only be annotated to the genus level. Interestingly, one of the 14 clones was annotated at the genus level as *Cyllamyces*. This adds weight to the current speculation as to whether *Cyllamyces* and *Caecomyces* are (Ozkose et al., 2001; Paul et al., 2018) or are not (Callaghan et al., 2015; Wang et al., 2017) distinct genera.

Within pure cultures, cloned sequences that were annotated differently varied in size relative to other clones (Table 1). As the 18S rRNA and 5.8S rRNA genic flanking regions were consistent in size, the variation in the amplicon size was associated only with the ITS1 region. However, in *N. frontalis* RE1 the large range in ITS1 size did not result in different annotations. This likely is a reflection of size differences in the *N. frontalis* RE1 clones being due to insertions rather than deletions within the ITS1 region. These findings highlight the need to sequence multiple clones from individual pure cultures to further refine current ITS1 based taxonomic frameworks for anaerobic fungi (Koetschan et al., 2014; Paul et al., 2018). For example, based on the full-length ITS1 sequences in the clone libraries, a 98% identity cut-off for clade (this study) or species equivalent (Paul et al., 2018) seems reasonable based on the average identity value within each clone library (Supplementary Table S2). However, when the minimum identity is considered within each clone library, then this cut-off value is only valid for one of the five pure cultures (*Piromyces* sp. CaDo16a). This is a limitation with the use of ITS1 as a taxonomic marker that cannot be easily circumvented, particularly when interpreting sequence data from cultivation independent analysis of environmental samples.

**FIGURE 1:**
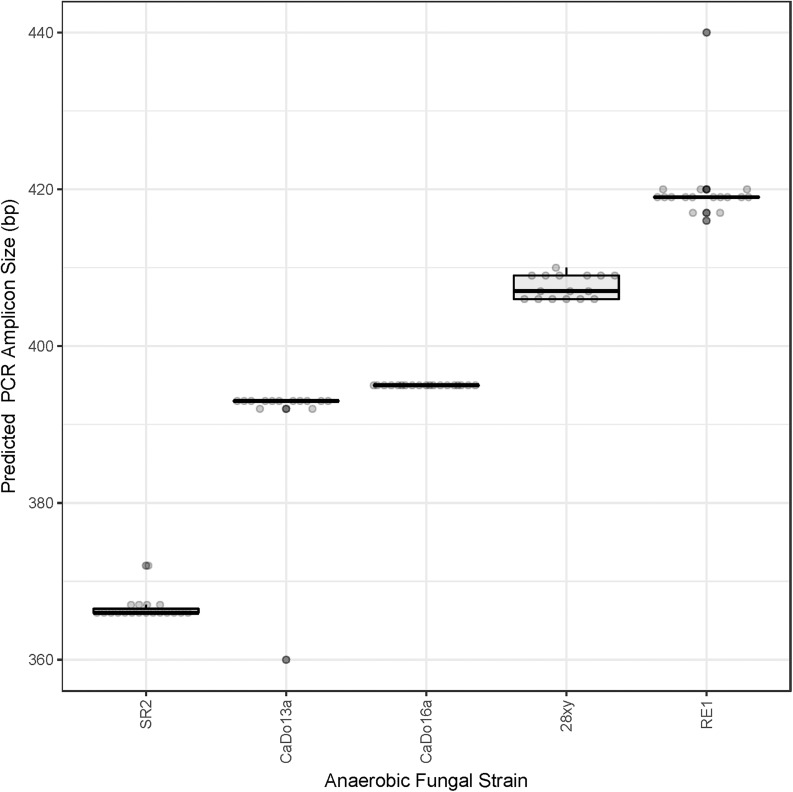
Prediction of the barcoded amplicon sizes. Clone library sequences (see Table 1) were used to predict *in silico* the amplicon size (excluding UniTag adapters and barcodes) that would be generated with the barcoded amplicon primers for each of the pure cultures: *N. frontalis* RE1 (*n* = 17), *Orpinomyces* sp. SR2 (*n* = 19), *Piromyces* sp. CaDo16a (*n* = 19), *Caecomyces* sp. CaDo13a (*n* = 14), and *Anaeromyces* sp. 28xy (*n* = 16).

All of the cloned sequences fully matched the primers used for the HTS. The clone library sequence data was used to predict the sizes of the amplicons that would be theoretically generated using HTS (Figure 1). The ITS1 region size range of the pure cultures (200–279 bases) was representative of the size range of the full-length ITS1 region sequences present within the AF-ITS1 database (192–282 bases).

### High-Throughput Sequence Analysis of the ITS1 Region of Anaerobic Fungal Pure Cultures in Terms of OTUs and Their Taxonomy

After processing of anaerobic fungal Illumina HiSeq data, the average number of reads per pure culture sample was 391,807 (SD 149,721) (Supplementary Table S1). Analysis of the pure culture HTS data indicated that the number of OTUs detected was generally consistent among sample replicates (*n* = 3), but varied greatly among the cultures: *Anaeromyces* sp. 28xy (28–29 OTUs), *Orpinomyces* sp. SR2 (14–15 OTUs), *N. frontalis* RE1 (12 OTUs), *Piromyces* sp. CaDo16a (3–7 OTUs), and *Caecomyces* sp. CaDo13a (3–5 OTUs). The number of OTUs was mostly higher (e.g., *Anaeromyces* sp. 28xy, *Orpinomyces* sp. SR2, *N. frontalis* RE1, and *Piromyces* sp. CaDo16a), but in one case lower (*Caecomyces* sp. CaDo13a), than the number of unique sequences detected in clone libraries (Table 1). More OTUs being detected was expected due to the increased sequencing depth (>10^4^ × higher coverage per pure culture) of the HTS method compared to the clone libraries. However, the detection of fewer OTUs was unexpected. The reason for this occurring with *Caecomyces* sp. CaDo13a was that a SNP was present in the partial 5.8S rRNA gene in an area that was not included in the barcoded amplicon. Due to this, the two different sequence types could not be distinguished using amplicon sequencing and resulted in a lower number of OTUs compared to the clone library.

**FIGURE 2:**
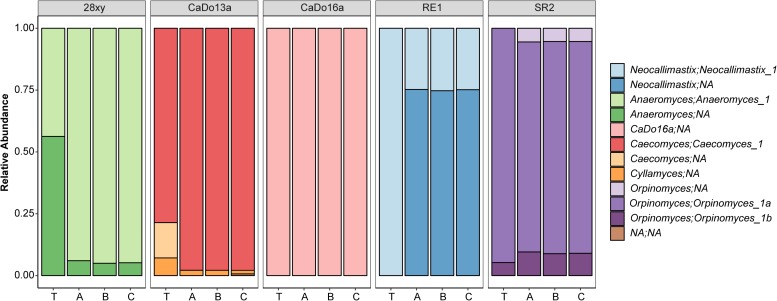
Taxonomic annotation of anaerobic fungal pure cultures based on high-throughput sequencing. The five different pure cultures (indicated by strain name) replicated by library (A, B, and C) are shown compared to the theoretical relative abundance (T), which was determined based on the corresponding clone library data (see Table 1). Annotations are indicated in terms of both genus and uncultured genus- or species-level clade. NA indicates that the clade or genus level could not be annotated within the family *Neocallimastigaceae*.

When summarized at the clade level, the BLAST based annotation of the OTUs was not always consistent with that predicted from the corresponding clone libraries (Figure 2). In two of the pure cultures, *N. frontalis* RE1 and *Orpinomyces* sp. SR2, fewer OTUs could be reliably assigned to the clade level compared to the clone libraries. Both of these cultures had more OTUs detected compared to the number of unique sequences in the corresponding clone libraries. In *Anaeromyces* sp. 28xy, the opposite was observed with a greater proportion of the OTUs that could be reliably assigned to the clade level compared to the clone library. The opposite differences in response between these genera is likely to be due to differences in terms of where variation between ITS1 copies is located within the ITS1 region, as the ITS1 region was only partially sequenced in the barcoded amplicons compared to being fully sequenced in the clone libraries. However, in all of the cultures the annotation at the genus level was consistent with that of the clone libraries. With *Caecomyces* sp. CaDo13a, the relative abundance of the *Caecomyces* and *Cyllamyces* genus annotations differed compared to that determined for the clone libraries. The higher sequencing depth with the HTS method is likely the reason for this, as the change in relative abundance is the opposite of what would be expected if the smaller sized *Cyllamyces* OTU was preferentially amplified (Figure 1 and Table 1).

### Assessment of Accuracy of High-Throughput Sequencing of the ITS1 Region of Anaerobic Fungi Using Mock Communities

Four mock communities (Mock_1 to Mock_4) that differed in composition were prepared. In general, these mock communities were representative of the anaerobic fungal community composition previously reported in the herbivore gut (Liggenstoffer et al., 2010). Mock_1 and Mock_2 were prepared using similar amounts of PCR amplicons from either two or five of the pure cultures, respectively. Mock_3 and Mock_4 were both composed of DNA from all five of the pure cultures, but in different proportions compared to Mock_2. Mock_3 had staggered proportions of each of the five pure cultures, whereas Mock_4 had several of the pure cultures at low abundances (i.e., 1, 0.1, and 0.01%). These four mock communities were then used for HTS in order to assess the accuracy of the method when applied to samples differing in complexity and diversity.

**FIGURE 3:**
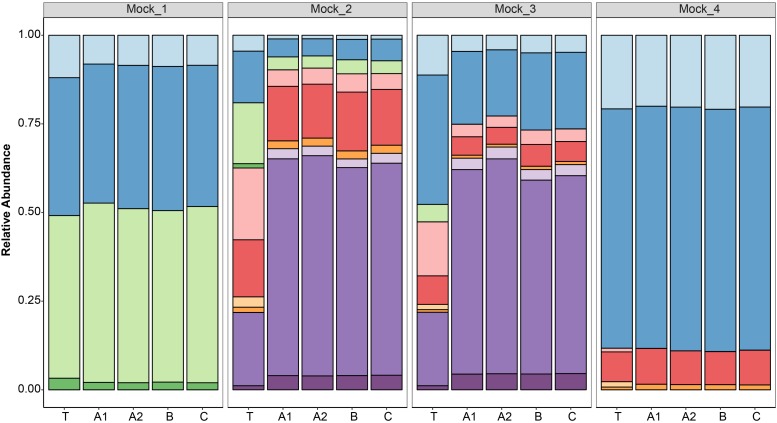
Taxonomic annotation of four defined anaerobic fungal mock communities (Mock_1 to Mock_4) based on high-throughput sequencing. Replicates were sequenced in three different libraries (A, B, and C), and duplicates were also prepared for one library (A1 and A2). The results of the replicated samples were compared to the relative abundances of the theoretical mock communities (T). The color key for the taxonomic annotations is the same as Figure 2.

**FIGURE 4:**
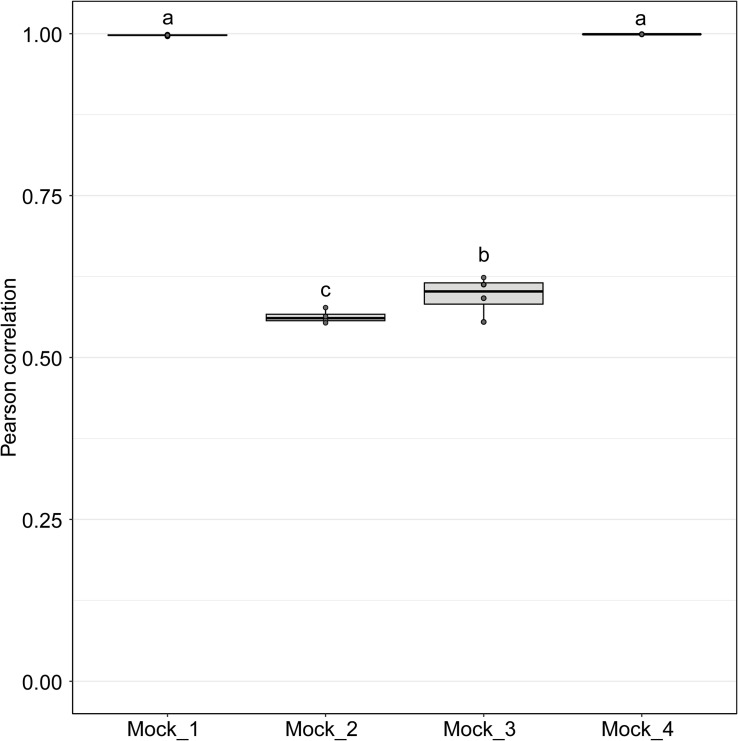
Pearson correlation values between sequenced mock communities (Mock_1 to Mock_4) and the corresponding theoretical mock communities. A value of 1 indicates a perfect match to the corresponding theoretical mock community. Mock communities with different letters significantly differ from each other in terms of Pearson correlation values (*P* < 0.001).

After data processing, the average number of reads per mock community sample was 211,817 (SD 58,041) (Supplementary Table S1). All the replicates (*n* = 4) of the sequenced mock communities generated similar profiles (Figure 3). Mock_1 and Mock_4 compared well to the theoretical composition of the corresponding mock community (Figure 4). However, in Mock_4 there was no OTU associated with *CaDo16a*; NA or *Caecomyces*; NA detected despite it being present at 1.1 and 1.5%, respectively, in the theoretical mock community (Table 2). This seems to contradict the detection of *Cyllamyces*; NA which was present at 0.8% in the theoretical mock community (Table 2). These observations indicate that the taxon detection limit of the method is not a “hard-line,” and is influenced by something other than the 0.6% minimum abundance threshold used during data processing. The high number of PCR cycles used to generate the barcoded amplicon, as with other studies (Liggenstoffer et al., 2010; Kittelmann et al., 2013), may offer a potential explanation for this. Under these conditions, minor taxa can be underrepresented if preferential amplification occurs or other templates are more abundant.

**TABLE 2:** Comparison of the percentage deviation^a^ of sequenced mock communities (*n* = 4) relative to the theoretical mock communities.

**Taxonomic Annotation^b^ (Genus; Clade)**	**Mock_1**		**Mock_2**		**Mock_3**		**Mock_4**	
	**Theor. Rel. Abund.**	**Deviation (SD)**	**Theor. Rel. Abund.**	**Deviation (SD)**	**Theor. Rel. Abund.**	**Deviation (SD)**	**Theor. Rel. Abund.**	**Deviation (SD)**
*Neocallimastix*; *Neocallimastix_1*	0.120	−29.2 (2.28)	0.045	−76.0 (1.66)	0.112	−58.9 (3.32)	0.208	−1.99 (1.77)
*Neocallimastix*; NA	0.389	2.9 (1.67)	0.146	−62.7 (4.07)	0.365	−43.4 (3.91)	0.675	1.49 (0.32)
*Anaeromyces*; *Anaeromyces_1*	0.459	7.8 (2.04)	0.172	−78.6 (1.52)	0.049	ND^∗^	0.000	–
*Anaeromyces*; NA	0.033	−37.1 (2.56)	0.012	ND^∗^	0.000	–	0.000	–
CaDo16a; NA	–	–	0.202	−76.8 (1.52)	0.152	−76.4 (2.36)	0.011	ND^∗^
*Caecomyces*; *Caecomyces_1*	–	–	0.161	−2.4 (3.83)	0.081	−32.7 (7.25)	0.084	15.5 (4.10)
*Caecomyces*; NA	–	–	0.029	ND^∗^	0.015	ND^∗^	0.015	ND^∗^
*Orpinomyces*; NA	–	–	–	D#	–	D#	–	–
*Orpinomyces*; *Orpinomyces_1a*	–	–	0.207	192.3 (7.30)	0.207	176.0 (12.44)	0.000	–
*Orpinomyces*; *Orpinomyces_1b*	–	–	0.011	248.2 (6.67)	0.012	289.9 (5.53)	0.000	–
*Cyllamyces*; NA	–	–	0.015	54.7 (2.51)	0.007	17.8 (8.04)	0.008	91.64 (9.98)

*^*a*^A deviation value of 0% indicates an identical match to the theoretical mock community. Mean deviation values are shown along with SD (in parentheses). ^*b*^Taxa in the table are arranged by decreasing amplicon size (as predicted from the clone library data), and NA indicates that the clade level could not be annotated. ^∗^ND indicates that the taxon was not detected in the sequenced mock community although it was present in the analyzed theoretical mock community. ^#^D indicates that the taxon was detected in the sequenced mock community (Figure 3), as well as the HiSeq sequenced pure cultures (Figure 2), but not present in the theoretical mock community (which was derived from the clone library data).*

**FIGURE 5:**
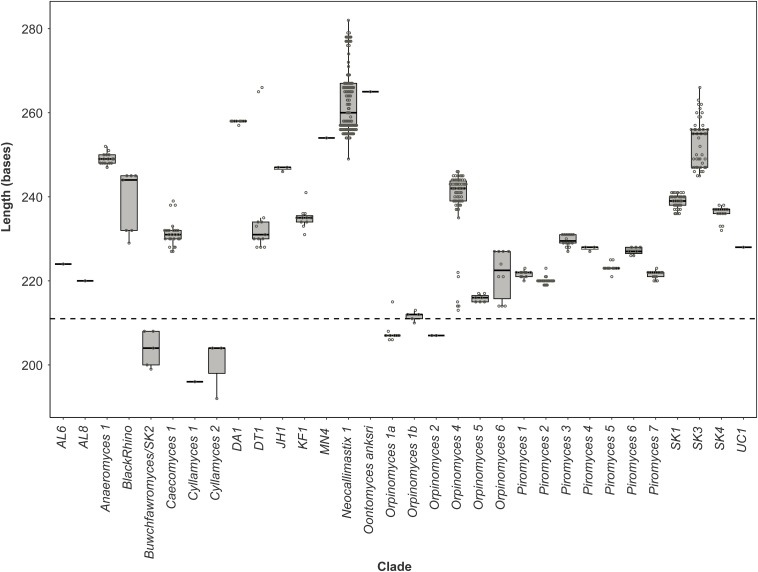
Size distribution of the anaerobic fungal ITS1 region. Boxplot of the size distribution of the full-length ITS1 region sequences present in the anaerobic fungal ITS1 database (version 3.3) that are associated with named clades. The dashed line indicates the size from which preferential amplification was observed in the mock communities (i.e., 211 bases is the ITS1 region length for the *Orpinomyces* sp. SR2 clone that was annotated as *Orpinomyces* 1b).

Pearson correlation values between the sequenced and theoretical mock communities were significantly higher for Mock_1 and Mock_4 compared to both Mock_2 and Mock_3 (*P* < 0.001) (Figure 4). Pearson correlation values for Mock_3 were also significantly higher than for Mock_2 (*P* < 0.001) (Figure 4). A significant difference between Mock_2 and Mock_3 compared to Mock_1 and Mock_4 (*P* < 0.001) was also found using weighted UniFrac distances (Supplementary Figure S2). In both Mock_2 and Mock_3 the relative abundances of *Orpinomyces*; *Orpinomyces 1a*, *Orpinomyces*; *Orpinomyces 1b*, and *Cyllamyces*; NA were much higher than expected (Figure 3 and Table 2). These three taxa represent the smallest of the barcoded amplicons predicted from the clone library data (Figure 1 and Table 1). Therefore, it is speculated that their higher relative abundance may be due to preferential amplification of these smaller amplicons during PCR. Analysis of the ITS1 region size in the AF-ITS1 database indicated that five of the 32 named clades were the same size or smaller than *Orpinomyces 1b* (Figure 5). Discrimination against longer PCR products has been previously reported when universal fungal primers were used for the entire ITS region (Ihrmark et al., 2012). In another study, no evidence of size bias in the ITS1 region was found when a mock community was analyzed using universal fungal primers, however, it was not stated what ITS1 size range the mock community represented (Tedersoo et al., 2015).

As amplicon sequencing data is inherently compositional, measurements of individual taxa are not independent (Gloor et al., 2017). Therefore, if the relative abundance of several taxa is higher than expected, the relative abundance of others is lower. This was clearly the case for Mock_2 and Mock_3. In Mock_2, taxa belonging to *Neocallimastix*, *Anaeromyces*, and CaDo16a were 0.6–0.8 fold lower than expected. In Mock_3, *Neocallimastix* and CaDo16a taxa were 0.4–0.8 fold lower than expected, and *Anaeromyces* was not detected at all despite accounting for 4.9% of the theoretical mock community.

From the poor match of Mock_2 and Mock_3 to the theoretical mocks, relative to Mock_1 and Mock_4, it can be concluded that the accuracy of the method is influenced by sample community composition. Consequently, there is a need to develop an alternative taxonomic marker for anaerobic fungi and associated curated database to ensure accurate analysis of environmental samples. In general, it has been reported that the ITS2 region is similar (Blaalid et al., 2013) or better (Yang et al., 2018) than the ITS1 region as a taxonomic marker for the fungal kingdom. Tuckwell et al. (2005) also showed that anaerobic fungal subgroups identified using ITS2 were broadly the same as subgroups identified using ITS1. However, in some cases Tuckwell et al. (2005) found for individual cultures sequence differences in the ITS1 region but not the ITS2 region, and vice versa. As a consequence of this, and the limited amount of ITS2 sequence data available for anaerobic fungi, it is perhaps not surprising that the anaerobic fungal research community has focused its attention on the 28S rRNA gene as an alternative to ITS1 (Edwards et al., 2017).

For anaerobic fungi, the D1/D2 region of the 28S rRNA gene appears to have a taxonomic resolution similar to the ITS1 region (Wang et al., 2017). As such, it has the potential to generate a more stable phylogenetic backbone for anaerobic fungi than ITS1 due to its more conserved size and, therefore, more limited heterogeneity within individual cultures. Anaerobic fungal specific primers targeting the D1/D2 region of the 28S rRNA gene have been developed (Dollhofer et al., 2016), and also used in conjunction with clone libraries to study the anaerobic fungal community composition of environmental samples (Dollhofer et al., 2017). However, reference sequences of this region for previously characterized taxa are currently limited (Wang et al., 2017). There is also a challenge in terms of how to relate 28S rRNA gene sequences to the uncultivated genus- or species level clades that have only been characterized to date based on environmentally derived ITS1 region sequences. Furthermore, contrasting findings have recently been reported when ITS1 and 28S rRNA gene clone libraries were both used to analyze anaerobic fungi in an environmental sample (Mura et al., 2018). Therefore, for now at least, it is likely that ITS1 will still be used to assess anaerobic fungal diversity and community structure in environmental samples until an alternative taxonomic marker, and associated taxonomic scheme and database (analogous to that currently available for ITS1), has been developed and evaluated.

## Conclusion

The findings of this study indicate that whilst HTS of the ITS1 region of anaerobic fungi can be used for environmental sample analysis, e.g., to detect differences between host species, diets, treatments groups etc., the accuracy of the method is influenced by sample community composition. Furthermore, ambiguity in the annotation of sequences within pure cultures due to ITS1 heterogeneity reinforces the limitations of the ITS1 region for the taxonomic assignment of anaerobic fungi. In order to overcome these issues, there is a need to develop an alternative taxonomic marker for anaerobic fungi.

## Data Availability Statement

The datasets generated for this study can be found in the NCBI database (MK036660-MK036676, MK036677-MK036695, MK036696-MK036714, MK036715-MK036728, and MK036729-MK036744) and the European Nucleotide Archive (PRJEB2913).

## Author Contributions

JE initiated the study, participated in the study design, conducted the lab work, analyzed and interpreted the data, drafted the manuscript, and obtained funding. GH and HS participated in the study design, interpretation of data, and drafting of the manuscript. BN and SK were involved in data analysis and interpretation, and drafting of the manuscript. All authors read and approved the final manuscript.

## Conflict of Interest

SK was an employee with the Wilmar International Limited. The remaining authors declare that the research was conducted in the absence of any commercial or financial relationships that could be construed as a potential conflict of interest.

## Footnotes

1 http://download.systemsbiology.nl/ngtax/

2 www.anaerobicfungi.org

3 http://download.systemsbiology.nl/ngtax/databases/

4 https://gitlab.com/wurssb/gen_fake_mocks/tree/master/paper_data/TestMock_150_06.biom

5 https://gitlab.com/wurssb/gen_fake_mocks/master/paper_data/otuseq_export.py

6 https://gitlab.com/wurssb/gen_fake_mocks/tree/master/paper_data/TestMock_150_06_TAX_FINAL_hdf5.biom

## Abbreviations

AF-ITS1: anaerobic fungal ITS1
HTS: high throughput sequencing
ITS1: internal transcribed spacer 1
NTC: non-template control
OTU: operational taxonomic unit
PCR: polymerase chain reaction
PE: paired end
SD: standard deviation
SNP: single nucleotide polymorphism.
