# Supplementary material for: Assessment of the Accuracy of High-Throughput Sequencing of the ITS1 Region of Neocallimastigomycota for Community Composition Analysis
Source: Front Microbiol. 2019 Oct 18;10:2370. doi: 10.3389/fmicb.2019.02370 (PMC6813465; doi:10.3389/fmicb.2019.02370)
Supplement: Supplementary file 1 [file Data_Sheet_1.pdf]

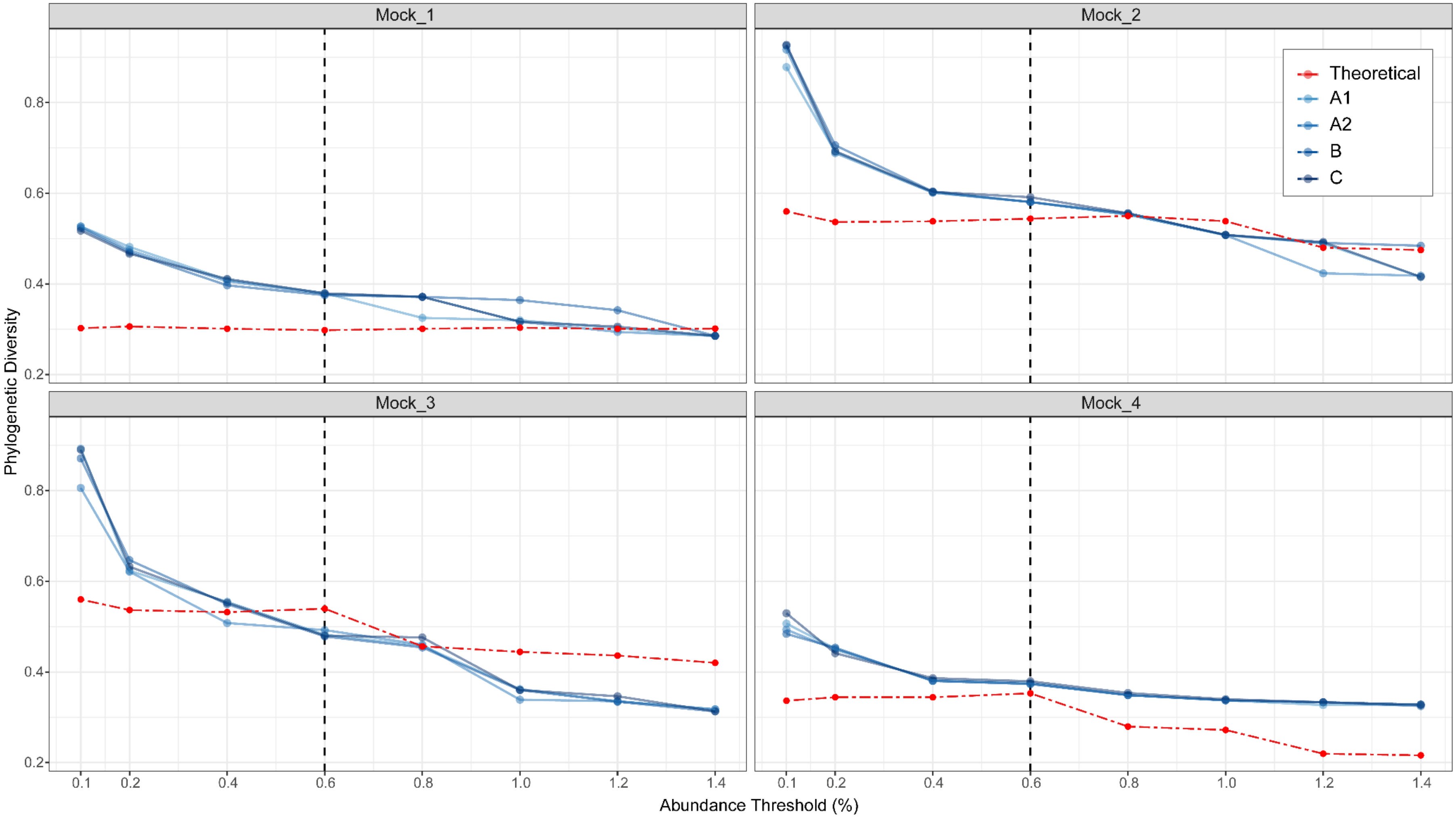


**Supplementary Figure 1.** Optimization of NG-Tax minimum percentage threshold for anaerobic fungal data analysis. Phylogenetic diversity[a] was calculated for each sequenced mock community (i.e. Mock_1 to Mock_4) using sample replicates (A1, A2, B and C) analyzed with different minimum abundance thresholds in NG-Tax. Theoretical 'perfect' mock community data (Theoretical) at a read depth of 200,000 reads was also analyzed in the same manner and is depicted with the red dashed line. Each mock community showed a different

optimal threshold where the theoretical and sequenced data intersected, trending towards higher thresholds with increased phylogenetic diversity. A threshold of 0.6% was defined as optimal after taking into account that biological diversity might exceed these artificial communities.

[a]Faith, D.P. (1994) Phylogenetic pattern and the quantification of organismal biodiversity. Philos Trans R Soc Lond B Biol Sci. 345:45-58.

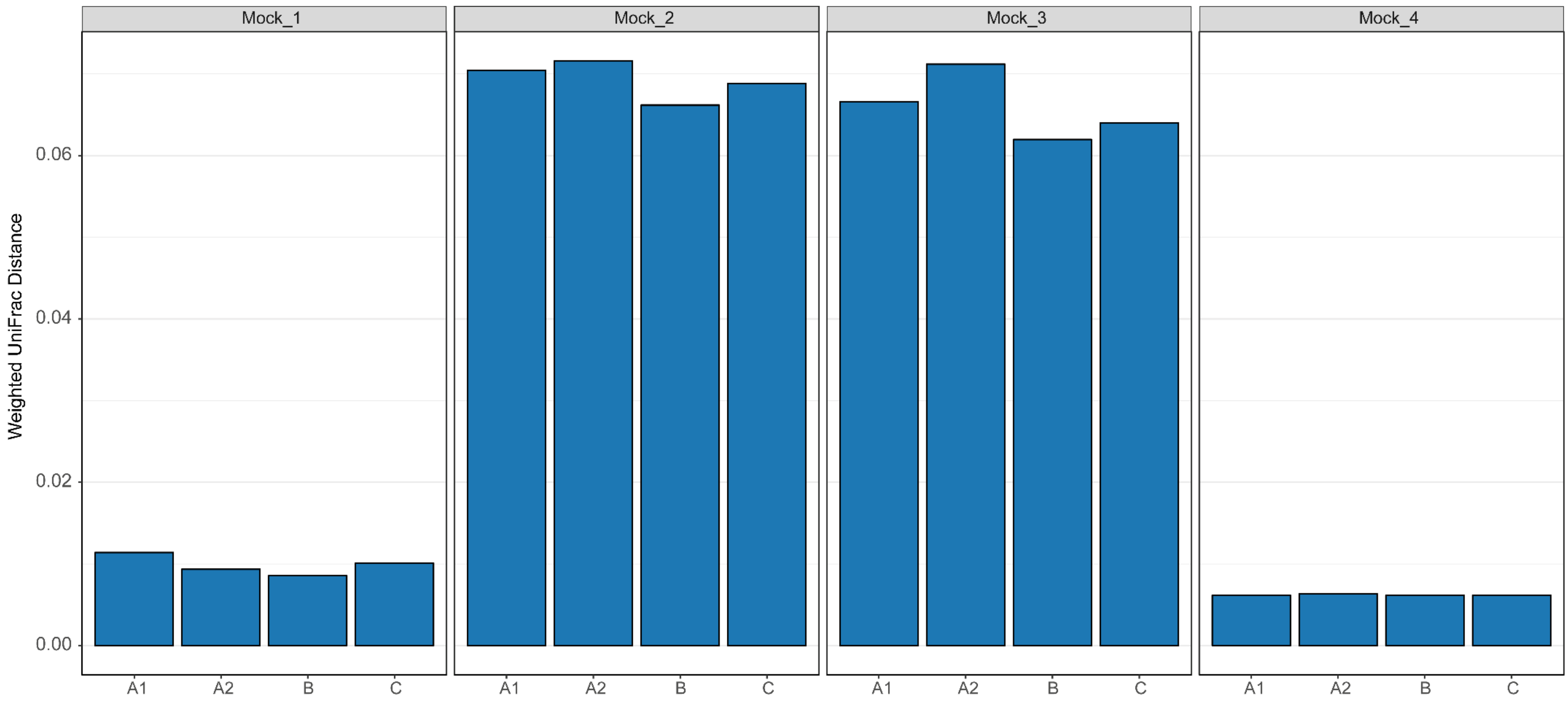


**Supplementary Figure 2.** Comparison of the weighted UniFrac distances between sequenced mock communities (Mock_1 to Mock_4) and the corresponding theoretical mock communities. Mock community replicates were sequenced in three different libraries (A, B, and C), and duplicates were also prepared for one library (A1 and A2). A distance of 0 indicates a perfect match to the corresponding theoretical mock community.

**Supplementary Table 1.** Details of sample barcodes, read counts and the associated library files for the high-throughput sequenced samples.

| Unique Sample ID | Sample | Read Count | Read Count After NTCs OTUs Removed[#] | Barcode Sequence | Replicate | Library Code | Library File |
|---|---|---|---|---|---|---|---|
| RE1 | RE1 | 254359 | 254359 | CATCAGTT | A | 1 | Lib1_FWD.fastq.gz, Lib1_REV.fastq.gz |
| SR2 | SR2 | 651495 | 651495 | ACCGGAAT | A | 1 | Lib1_FWD.fastq.gz, Lib1_REV.fastq.gz |
| CaDo16a | CaDo16a | 390987 | 390987 | TACCTACT | A | 1 | Lib1_FWD.fastq.gz, Lib1_REV.fastq.gz |
| CaD013a | CaD013a | 523577 | 523577 | TGCCTCTC | A | 1 | Lib1_FWD.fastq.gz, Lib1_REV.fastq.gz |
| 28xy | 28xy | 346951 | 346951 | ACTGCTCT | A | 1 | Lib1_FWD.fastq.gz, Lib1_REV.fastq.gz |
| PC_Neg | PC_NTC_A | 18587 | 0 | ACGAGCGA | - | 1 | Lib1_FWD.fastq.gz, Lib1_REV.fastq.gz |
| RE1_a | RE1 | 267851 | 267851 | TACCTACT | B | 2 | Lib2_FWD.fastq.gz, Lib2_REV.fastq.gz |
| SR2_a | SR2 | 586722 | 586722 | TGCCTCTC | B | 2 | Lib2_FWD.fastq.gz, Lib2_REV.fastq.gz |
| CaDo16a_a | CaDo16a | 345758 | 345758 | ACTGCTCT | B | 2 | Lib2_FWD.fastq.gz, Lib2_REV.fastq.gz |
| CaD013a_a | CaD013a | 281869 | 275756 | ACGAGCGA | B | 2 | Lib2_FWD.fastq.gz, Lib2_REV.fastq.gz |
| 28xy_a | 28xy | 203892 | 203892 | CGTACTGA | B | 2 | Lib2_FWD.fastq.gz, Lib2_REV.fastq.gz |
| RE1_b | RE1 | 299793 | 299793 | ACCGGAAT | C | 3 | Lib3_FWD.fastq.gz, Lib3_REV.fastq.gz |
| SR2_b | SR2 | 661383 | 661383 | TACCTACT | C | 3 | Lib3_FWD.fastq.gz, Lib3_REV.fastq.gz |
| CaDo16a_b | CaDo16a | 438999 | 438999 | TGCCTCTC | C | 3 | Lib3_FWD.fastq.gz, Lib3_REV.fastq.gz |
| CaD013a_b | CaD013a | 398153 | 380520 | ACTGCTCT | C | 3 | Lib3_FWD.fastq.gz, Lib3_REV.fastq.gz |
| 28xy_b | 28xy | 249065 | 249065 | ACGAGCGA | C | 3 | Lib3_FWD.fastq.gz, Lib3_REV.fastq.gz |
| PC_Neg_ab | PC_NTC_BC | 26007 | 1048 | CGTACTGA | - | 3 | Lib3_FWD.fastq.gz, Lib3_REV.fastq.gz |
| AF1_a | Mock_1 | 162131 | 162131 | GAAGCTCG | A1 | 1 | Lib1_FWD.fastq.gz, Lib1_REV.fastq.gz |
| AF2_a | Mock_2 | 272322 | 272322 | AAGGTAAG | A1 | 1 | Lib1_FWD.fastq.gz, Lib1_REV.fastq.gz |
| AF3_a | Mock_3 | 300356 | 300356 | ATGCGATT | A1 | 1 | Lib1_FWD.fastq.gz, Lib1_REV.fastq.gz |
| AF4_a | Mock_4 | 251933 | 251933 | AAGATCGT | A1 | 1 | Lib1_FWD.fastq.gz, Lib1_REV.fastq.gz |
| AF_Neg_a | AF_NTC_A1 | 124156 | 12283 | ATATAGGA | - | 1 | Lib1_FWD.fastq.gz, Lib1_REV.fastq.gz |

| | | | | | | | |
|---|---|---|---|---|---|---|---|
| AF1_b | Mock_1 | 143125 | 143125 | TATATTGA | A2 | 1 | Lib1_FWD.fastq.gz, Lib1_REV.fastq.gz |
| AF2_b | Mock_2 | 159747 | 159747 | TCCAGCTC | A2 | 1 | Lib1_FWD.fastq.gz, Lib1_REV.fastq.gz |
| AF3_b | Mock_3 | 156386 | 156386 | GCTTGATG | A2 | 1 | Lib1_FWD.fastq.gz, Lib1_REV.fastq.gz |
| AF4_b | Mock_4 | 203536 | 203536 | GTAAGAAG | A2 | 1 | Lib1_FWD.fastq.gz, Lib1_REV.fastq.gz |
| AF_Neg_b | AF_NTC_A2 | 126505 | 6264 | GAACGCTG | - | 1 | Lib1_FWD.fastq.gz, Lib1_REV.fastq.gz |
| AF_NTC | AF_NTC_A2a | 2 | 0 | CTGACCGG | - | 1 | Lib1_FWD.fastq.gz, Lib1_REV.fastq.gz |
| AF1_b_rep | Mock_1 | 169880 | 169880 | GTAAGAAG | B | 2 | Lib2_FWD.fastq.gz, Lib2_REV.fastq.gz |
| AF2_b_rep | Mock_2 | 219134 | 219134 | GAACGCTG | B | 2 | Lib2_FWD.fastq.gz, Lib2_REV.fastq.gz |
| AF3_b_rep | Mock_3 | 276904 | 276904 | CTGACCGG | B | 2 | Lib2_FWD.fastq.gz, Lib2_REV.fastq.gz |
| AF4_b_rep | Mock_4 | 170148 | 170148 | CATCAGTT | B | 2 | Lib2_FWD.fastq.gz, Lib2_REV.fastq.gz |
| AF_Neg_b_rep | AF_NTC_B | 96719 | 4481 | ACCGGAAT | - | 2 | Lib2_FWD.fastq.gz, Lib2_REV.fastq.gz |
| AF1_c | Mock_1 | 120979 | 120979 | GCTTGATG | C | 3 | Lib3_FWD.fastq.gz, Lib3_REV.fastq.gz |
| AF2_c | Mock_2 | 276919 | 276919 | GTAAGAAG | C | 3 | Lib3_FWD.fastq.gz, Lib3_REV.fastq.gz |
| AF3_c | Mock_3 | 233418 | 233418 | GAACGCTG | C | 3 | Lib3_FWD.fastq.gz, Lib3_REV.fastq.gz |
| AF4_c | Mock_4 | 272162 | 272162 | CTGACCGG | C | 3 | Lib3_FWD.fastq.gz, Lib3_REV.fastq.gz |
| AF_Neg_c | AF_NTC_C | 141827 | 0 | CATCAGTT | - | 3 | Lib3_FWD.fastq.gz, Lib3_REV.fastq.gz |
| AF1_t | Mock_1_t | 96829 | 96829 | AAAAAAAA | - | 4 | Mock_1_1.fq.gz, Mock_1_2.fq.gz |
| AF2_t | Mock_2_t | 98815 | 98815 | CCCCCCCC | - | 5 | Mock_2_1.fq.gz, Mock_2_2.fq.gz |
| AF3_t | Mock_3_t | 100066 | 100066 | GGGGGGGG | - | 6 | Mock_3_1.fq.gz, Mock_3_2.fq.gz |
| AF4_t | Mock_4_t | 99894 | 99894 | TTTTTTTT | - | 7 | Mock_4_1.fq.gz, Mock_4_2.fq.gz |

[#] In order to decrease background noise in the data, read counts for operational taxonomic units (OTUs) that were present in the non-template controls (NTCs) were removed. Remaining reads in four of the seven NTCs were due to six OTUs that had a low read count/proportion only in the NTCs (i.e. <4,000 reads and <5%), and were otherwise only present in the samples that contained the pure culture which the OTU(s) related to.

**Supplementary Table 2.** Comparison of the identity of the full-length ITS1 region sequences within each pure culture clone library. All the cloned sequences were aligned using ClustalW version 2.1[b], and the genic flanking regions were trimmed off the alignment using GeneDoc version 2.6[c]. The start of the ITS1 region was defined based on the end of the 18S rRNA gene (GATCATTA). The end of the ITS1 region was defined based on the start of the 5.8S rRNA gene (CAACTTT). All the ITS1 sequences derived from the same pure culture were then directly compared using a sequence identity matrix generated with BioEdit version 7.2.5[d]. For each pure culture, the minimum and maximum identity values are reported along with average of all the values from the identity matrix, and the corresponding standard deviation (SD).

| Genus | Strain | Min | Max | Average (SD) |
|---|---|---|---|---|
| *Neocallimastix* | RE1 | 0.823 | 1.000 | 0.973 (0.053) |
| *Orpinomyces* | SR2 | 0.952 | 1.000 | 0.989 (0.012) |
| *Piromyces* | CaDo16a | 1.000 | 1.000 | 1.000 (0.000) |
| *Caecomyces* | CaDo13a | 0.700 | 1.000 | 0.950 (0.103) |
| *Anaeromyces* | 28xy | 0.952 | 1.000 | 0.978 (0.014) |

[b] Larkin, M. A., Blackshields, G., Brown, N. P., Chenna, R., McGettigan, P. A., McWilliam, H., et al. (2007). Clustal W and Clustal X version 2.0. Bioinformatics. 23, 2947-2948.
[c] Nicholas, K. B., Nicholas, H. B. (1997). GeneDoc: a tool for editing and annotating multiple sequence alignments. Distributed by the author.
[d] Hall, T. A. (1999) BioEdit: a user-friendly biological sequence alignment editor and analysis program for Windows 95/98/NT. Nucl. Acids Symp. Ser. 41, 95-9.
